# Intestinal microbiome profiles in broiler chickens raised without antibiotics exhibit altered microbiome dynamics relative to conventionally raised chickens

**DOI:** 10.1371/journal.pone.0301110

**Published:** 2024-04-03

**Authors:** Seyed Hossien Kairmi, Khaled Abdelaziz, Heidi Spahany, Jake Astill, David Trott, Blake Wang, Alice Wang, John Parkinson, Shayan Sharif

**Affiliations:** 1 Department of Pathobiology, Ontario Veterinary College, University of Guelph, Guelph, Ontario, Canada; 2 Department of Animal and Veterinary Sciences, Clemson University, Clemson, SC, United States of America; 3 Clemson University School of Health Research (CUSHR), Clemson, South Carolina, United States of America; 4 Wallenstein Feed & Supply Ltd, Wallenstein, Ontario, Canada; 5 Program in Molecular Medicine, Hospital for Sick Children, Toronto, Ontario, Canada; 6 Department of Biochemistry & Molecular Genetics, University of Toronto, Toronto, Ontario, Canada; University of Illinois, UNITED STATES

## Abstract

The present study was undertaken to profile and compare the cecal microbial communities in conventionally (CONV) grown and raised without antibiotics (RWA) broiler chickens. Three hundred chickens were collected from five CONV and five RWA chicken farms on days 10, 24, and 35 of age. Microbial genomic DNA was extracted from cecal contents, and the V4-V5 hypervariable regions of the 16S rRNA gene were amplified and sequenced. Analysis of 16S rRNA sequence data indicated significant differences in the cecal microbial diversity and composition between CONV and RWA chickens on days 10, 24, and 35 days of age. On days 10 and 24, CONV chickens had higher richness and diversity of the cecal microbiome relative to RWA chickens. However, on day 35, this pattern reversed such that RWA chickens had higher richness and diversity of the cecal microbiome than the CONV groups. On days 10 and 24, the microbiomes of both CONV and RWA chickens were dominated by members of the phylum Firmicutes. On day 35, while Firmicutes remained dominant in the RWA chickens, the microbiome of CONV chickens exhibited am abundance of Bacteroidetes. The cecal microbiome of CONV chickens was enriched with the genus *Faecalibacterium*, *Pseudoflavonifractor*, unclassified *Clostridium_IV*, *Bacteroides*, *Alistipes*, and *Butyricimonas*, whereas the cecal microbiome of RWA chickens was enriched with genus *Anaerofilum*, *Butyricicoccu*, *Clostridium_XlVb* and unclassified *Lachnospiraceae*. Overall, the cecal microbiome richness, diversity, and composition were greatly influenced by the management program applied in these farms. These findings provide a foundation for further research on tailoring feed formulation or developing a consortium to modify the gut microbiome composition of RWA chickens.

## Introduction

Subtherapeutic doses of antibiotics have long been used in conventional (CONV) poultry farms for improving growth performance and controlling various enteric pathogens. However, the growing public health concerns over the emergence and spread of multi-drug-resistant bacteria have led to a reduction of antibiotic use in poultry feed [1, 2]. As such, Canadian poultry producers have voluntarily taken steps to phase out the medically important antibiotics, including categories I, II, and III, from poultry feed [3–6]. This proactive initiative not only demonstrates the industry’s dedication to the responsible use of antibiotics but also marks a significant departure from traditional approaches to embrace programs focused on raising animals without the use of antibiotics, commonly referred to as Raised Without Antibiotics (RWA) programs [3–6].

It is thought that dietary antibiotics exert their growth-promoting effects by modulating the composition of the gut microbiota by limiting the growth of pathogenic bacteria that compete for micronutrients in the gut without suppressing the growth of beneficial microbes. In the absence of antibiotics, opportunistic pathogens inhabiting the chicken intestine may undergo uncontrolled proliferation and excessive secretion of toxins, leading to intestinal inflammation, reduced performance, and increased mortality [7]. This notion is consistent with the recent demonstration that antibiotic withdrawal from poultry feed was associated with increased outbreaks of *Clostridium perfringens*-induced necrotic enteritis (NE) in poultry farms [8–10].

Until recently, it was unclear how antibiotics selectively suppress the growth of pathogenic bacteria without causing perturbation of the gut microbiome. Recent advances in high-throughput next-generation sequencing (NGS) technologies have advanced our understanding of the microbiome composition and function and, more importantly, have aided in tracking the dynamic changes in the composition of the intestinal microbiome in response to dietary antibiotics and other additives [reviewed by 11, 12]. Indeed, NGS-based 16S rRNA gene sequencing of the intestinal microbial DNA has been used by several research groups, including our own, to investigate the influence of subtherapeutic and therapeutic doses of various antibiotics on the composition of the chicken intestinal microbiota [13]. In this context, supplementation of broiler chickens with various antibiotics, including virginiamycin, chlortetracycline, bacitracin methyl disalicylate, lincomycin, oxytetracycline di-hydrate, and tylosin phosphate, was found to be associated with a reduction in the abundance of pathogenic bacteria, such as *Escherichia coli* and *C*. *perfringens*. In contrast, no significant alterations in the microbiome diversity and the relative abundance of beneficial bacteria, particularly *Lactobacillus* spp., were observed [14]. Similarly, supplementing chicken diets with subtherapeutic doses of bacitracin methylene disalicylate, tylosin, and virginiamycin preferentially enriched the cecal microbiome with butyrate- and lactic acid-producing bacteria, which both have been known for their role in modulating the gut mucosal immunity and host metabolism. However, when supplemented at therapeutic doses, antibiotics, including bacitracin methylene disalicylate (BMD) and penicillin G potassium (PP), negatively impacted the richness, evenness, and diversity of the chicken cecal microbiota [13].

With parts of the poultry industry transitioning to RWA programs and the continuous improvement of management practices to prevent the introduction and spread of infectious diseases into poultry flocks, several strategies have been adopted as alternatives to antimicrobials. These strategies include feed and water additives, such as enzymes, acidified water, organic acids, essential oils, prebiotics, and probiotics. The effects of various antibiotics and antimicrobial alternatives on the intestinal microbiome composition have been experimentally investigated. Despite the role of these studies in elucidating differential changes in the intestinal microbial profiles in response to dietary supplements, there is limited information on whether the changes observed in experimental settings can be extrapolated to commercial settings, where various management procedures are applied. Understanding the changes induced by antimicrobial growth promoters (AGPs) on the intestinal microbial community and the impact of the withdrawal of AGPs under field conditions will help devise effective antimicrobial alternatives. Therefore, this study was conducted to profile and compare the dynamic changes in cecal microbial communities of broiler chickens raised on conventional and RWA programs at different stages of their life.

## Materials and methods

### Animals

Three-hundred mixed ROSS 708 broiler chickens were collected from ten Ontario farms, including five CONV and five RWA flocks, during the summer of 2018. Chickens in these farms received the same diet from Wallenstein Feed & Supply Ltd, Ontario, Canada. Table 1 shows the chemical composition of the diets received. Birds were housed in environmentally controlled facilities with temperatures maintained between 22°C and 33°C at placement and the relative humidity ranged from 50 to 70%. Temperatures were gradually lowered by 2°C to 3°C per week, aiming to achieve a target temperature of 22°C by day 35 of the study.

**Table 1:** Chemical composition (mean±SD) of experimental diets.

	Starter		Grower		Finisher	
Program^1^	RWA	CONV	RWA	CONV	RWA	CONV
Dry matter, %	86.84±0.01	87.39±0.02	86.49±0.02	87.59±0.01	86.75±0.04	87.49±0.06
Crude protein, %	22.89±0.02	24.47±0.01	17.98±0.009	19.67±0.15	16.74±0.01	17.80±0.09
Crude fat, %	3.31±0.03	4.56±0.03	3.23±0.17	3.57±0.13	3.57±0.28	3.60±0.05
Crude fibre, %	2.90±-0.03	3.19±0.02	2.66±0.01	2.97±0.03	2.54±0.05	2.85±0.06
Calcium total, %	0.85±0.00	0.83±0.00	0.70±0.00	0.75±0.00	0.65±0.00	0.70±0.00
Phosphorus total, %	0.59±0.004	0.59±0.004	0.51±0.01	0.50±0.004	0.47±0.003	0.48±0.00
Sodium, %	0.20±0.00	0.17±0.00	0.16±0.01	0.20±0.00	0.20±0.009	0.20±0.00
Chloride, %	0.26±0.00	0.22±0.00	0.22±0.01	0.28±0.00	0.28±0.01	0.28±0.00
Vomitoxin, ppm	0.81±0.12	0.71±0.01	0.85±0.13	0.17±0.01	0.86±0.05	1.28±0.47

^1^ Gut health management program: CONV, RWA, raised without antibiotics.

This study was conducted in accordance with the Canadian Council on Animal Care (CCAC) regulations and was approved by the Animal Care Committee at the University of Guelph (Animal Utilization Protocol # 3410).

### Sample collection and DNA extraction

Chickens (n = 10 per farm) were euthanized by CO2 inhalation at days 10, 24, and 35 of age, and their cecal contents were snap-frozen on dry ice and then stored at -80°C until further use [15]. DNA extraction was performed as described previously [13]. The quality and concentration of DNA samples were measured as described previously with a Nanodrop spectrophotometer (NanoDrop Technologies, Wilmington, DE) [13].

### DNA sequencing

The V4-V5 hypervariable regions of the 16S rRNA gene were amplified and sequenced with an Illumina MiSeq sequencer at the Integrated Microbiome Resource, Dalhousie University. The pair primer set used in the study was 515FB = GTGYCAGCMGCCGCGGTAA and 926R = CCGYCAATTYMTTTRAGTTT. A total of 5,384,859 sequences were produced, with an average of 19,717 sequences per sample.

### Sequence processing and bioinformatics analysis

Sequences were processed using Mothur (v.1.44.) [16], following the MiSeq SOP [17] as described previously [13]. Briefly, the forward and reverse reads were combined by generating make.contigs command. After removing non-assembled and merging duplicated sequences, screened sequences were used for alignment in the Silva 16S rRNA gene database v. 138 with align.seqs command. The V4-V5 regions of the 16S rRNA gene were selected and sequences that had ambiguous bases less than 412 bp or were not classified as bacteria or contained homopolymers of >9 bp were removed. The redundant aligned reads and the chimeric sequences were removed by unique.seqs and chimera.vsearch commands, respectively. Sequences were clustered into operation taxonomic units (OTUs) by dist.seqs (cutoff = 0.03) and cluster commands. The number of sequence reads assigned to each OTU in each sample was generated by make.shared command. The Ribosomal Database Project (RDP) classifier was used to assign the sequences in the bacterial taxonomy classifier. Mothur was used for all OTU-based, alpha, and beta diversity analyses. The nonmetric multidimensional scaling (NMDS) approach was performed using the Bray Curtis dissimilarity index to analyze the bacterial composition difference in the treatments. Statistical comparison of spatial separation on the NMDS were analyzed on community dissimilarity using permutational multivariate analysis of variance (PERMANOVA) using the Adonis function in the R’s Vegan package [v.3.5.1.] with 1,000 permutations. When all pairwise comparisons were significant using PERMANOVA, permutational analysis of multivariate dispersions (PERMDISP) was applied in the R’s Vegan package with the function betadisper [v.3.5.1.] to determine if such differences arose from differences in the dispersion of samples. Multiple testing corrections (PERMANOVA and PERMDISP tests) were performed using Benjimani-Hochberg procedure [18].

DESeq2 R package was used to define the significantly difference taxa among gut health management programs, including CONV and RWA.

## Results

### The impact of different rearing on the structure of the cecal microbiome

**Fig 1:**
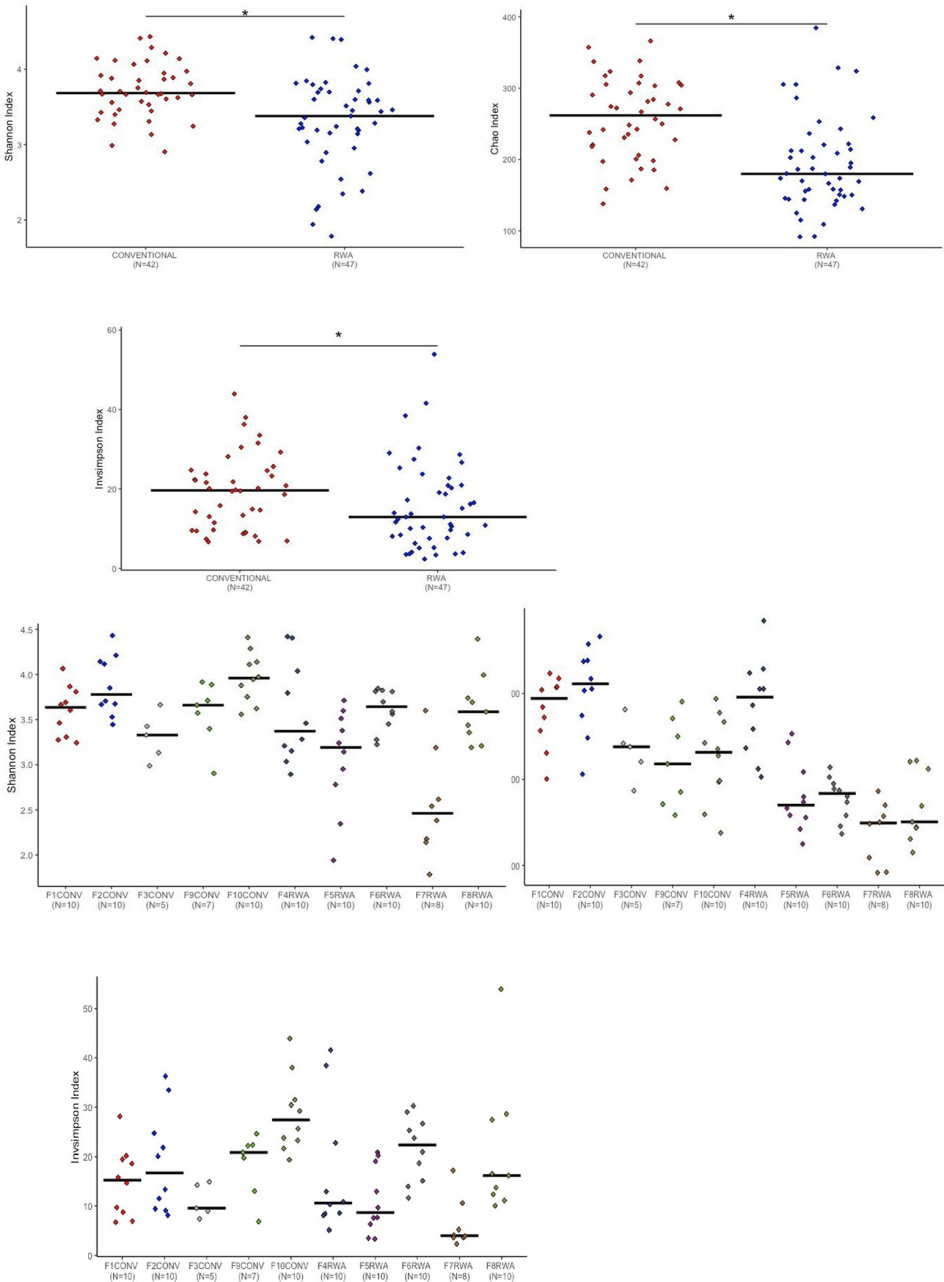
Measurement of alpha diversity of the different chicken management programs on day 10 of age by using Chao, Inverse Simpson and Shannon indexes or a diet containing therapeutical dosage of antibiotics and ionophores at conventional gut health program or raised without any antibiotic in the RWA farms. * indicates a significant difference at p<0.05 between CONV and RWA that were compared. n.s. indicates a non-significant difference at p<0.05 between CONV and RWA programs.

**Fig 2:**
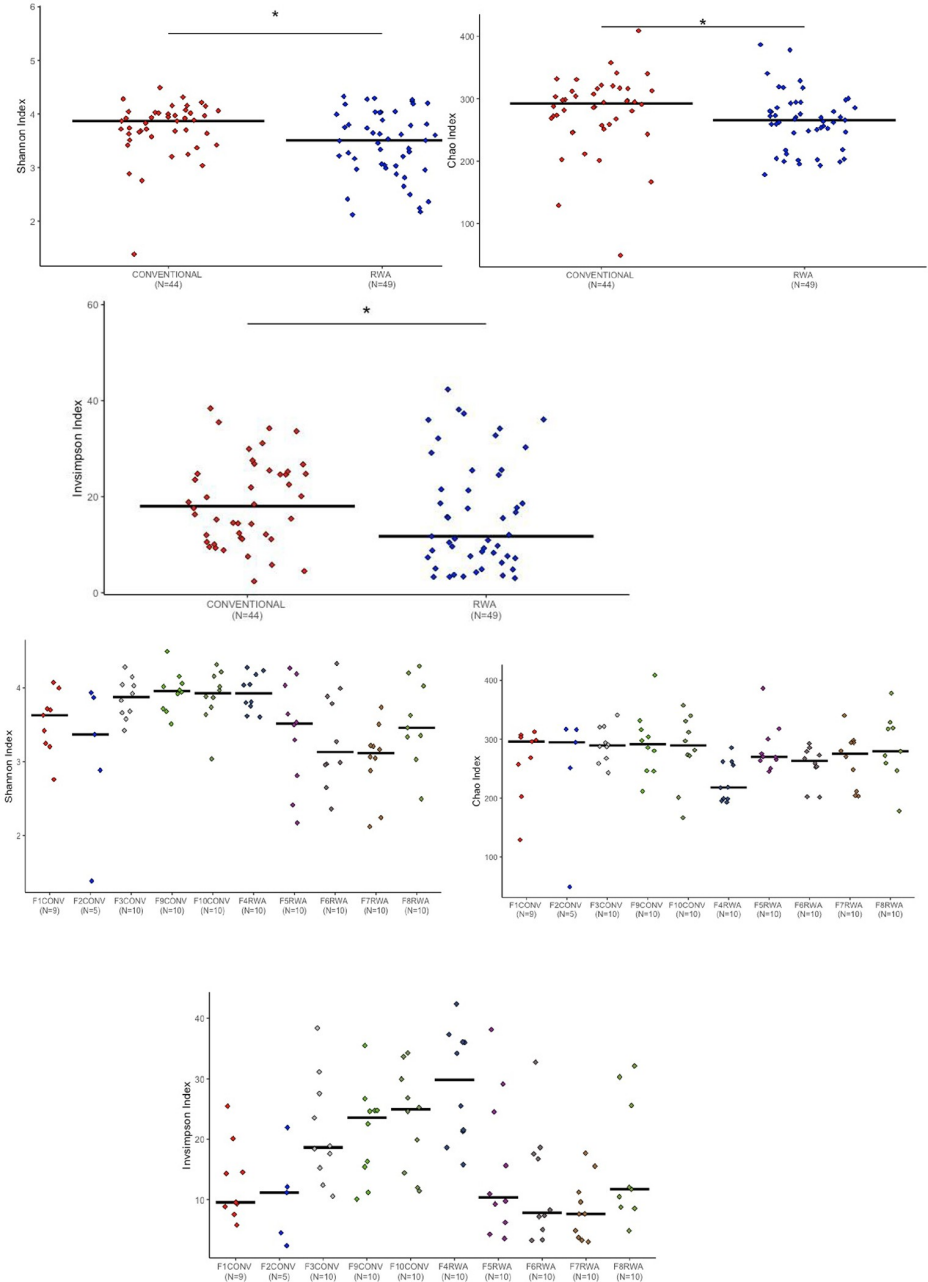
Measurement of alpha diversity of the different chicken management programs on day 24 of age by using Chao, Inverse Simpson and Shannon indexes or a diet containing therapeutical dosage of antibiotics and ionophores at conventional gut health program or raised without any antibiotic in the RWA farms. * indicates a significant difference at p<0.05 between CONV and RWA that were compared. n.s. indicates a non-significant difference at p<0.05 between CONV and RWA programs.

**Fig 3:**
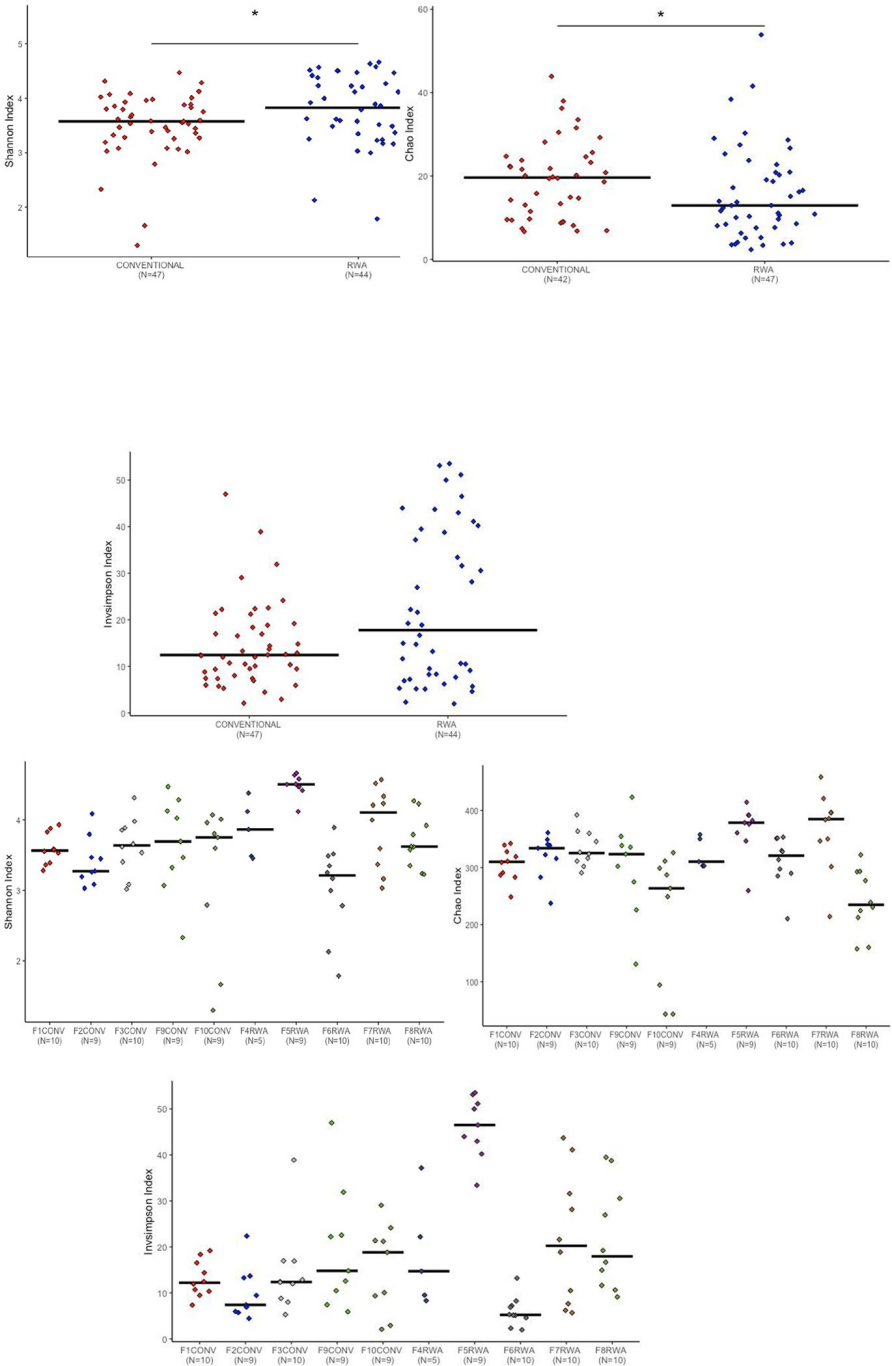
Measurement of alpha diversity of the different chicken management programs on day 10 of age by using Chao, Inverse Simpson and Shannon indexes or a diet containing therapeutical dosage of antibiotics and ionophores at conventional gut health program or raised without any antibiotic in the RWA farms. * indicates a significant difference at p<0.05 between CONV and RWA that were compared. n.s. indicates a non-significant difference at p<0.05 between CONV and RWA programs.

We initially applied three alpha diversity metrics to identify the significant diversity within the microbial community of the CONV and RWA birds. The results of alpha diversity of the cecal microbiome of CONV and RWA chickens on days 10, 24 and 35 are shown in Figs 1–3.

The sampling days were selected based on the typical approach of switching diets in the different stages of the bird’s growth. The microbial richness was measured with the Chao index and Shannon and Inverse Simpson indexes calculated the microbial diversity and evenness. Significant differences were observed in the Chao, Shannon and Inverse Simpson parameters between chickens reared on CONV and RWA programs on days 10 and 24 of age.

The conventionally raised chickens had a significantly higher cecal microbiome richness and diversity in comparison to chickens raised on the RWA program (P < 0.05, ANOVA) (Figs 1 and 2). On day 35 of age, the conventionally raised chickens had higher cecal microbial community richness, whereas RWA chickens had higher cecal microbial diversity (Fig 3). Overall, these results align with our previous study, which underscored the significant impact of gut health management programs on the composition of the gut microbiome [13].

### Beta diversity of the cecal microbiome

**Fig 4:**
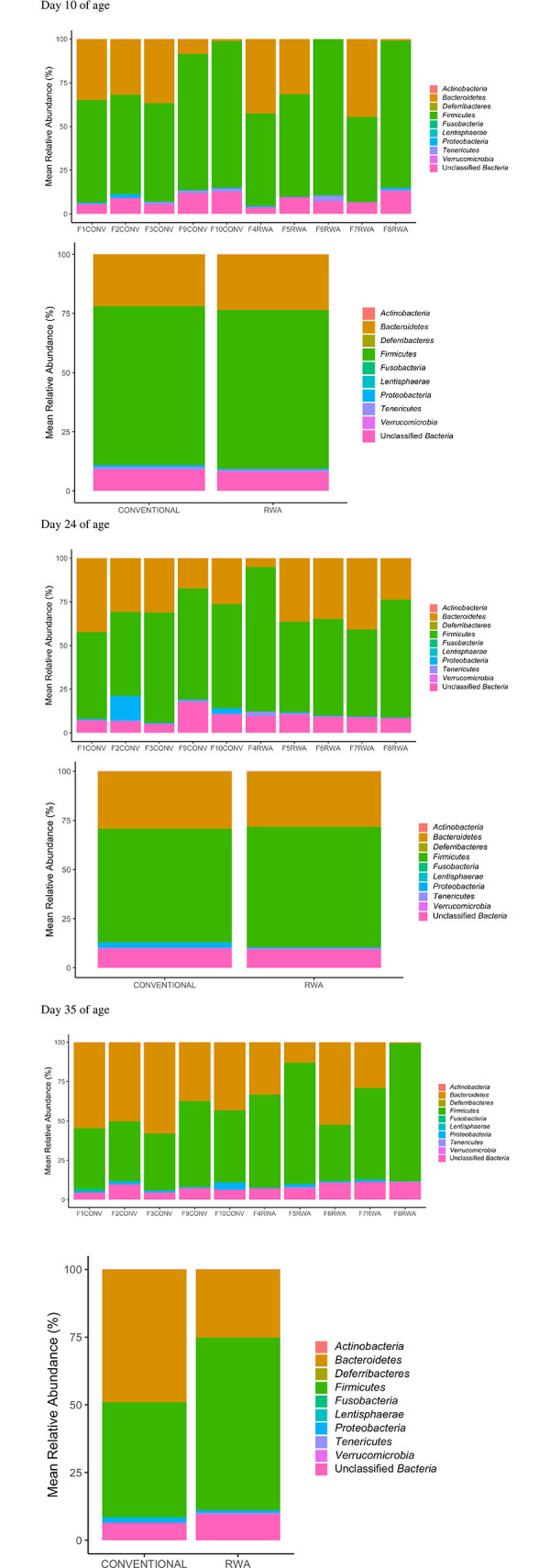
Microbial community differences in the cecal microbiome of the birds raised in the CONV and RWA groups during the growing phase: Starter (d 1 to 10 of age), grower (d 11 to 24 of age), and finisher (d 25 to 35 of age).

The composition of the cecal microbiome of CONV and RWA chickens at the phylum level on days 10, 24 and 35 of age is shown in Fig 4.

**Fig 5:**
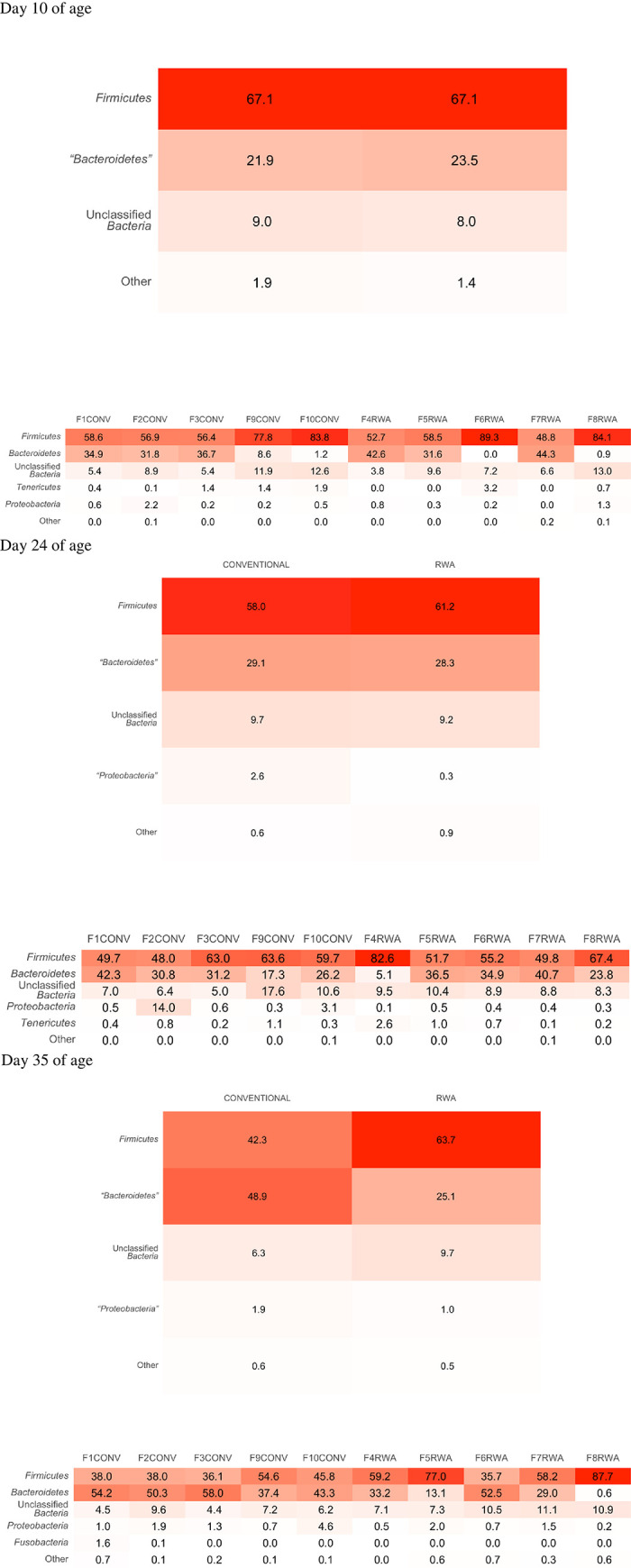
Heatmap comparing the relative abundance of difference in the phylum level in the cecal microbiome of birds raised in the CONV and RWA groups during the growing phase: Starter (d 1 to 10 of age), grower (d 11 to 24 of age), and finisher (d 25 to 35 of age).

**Fig 6:**
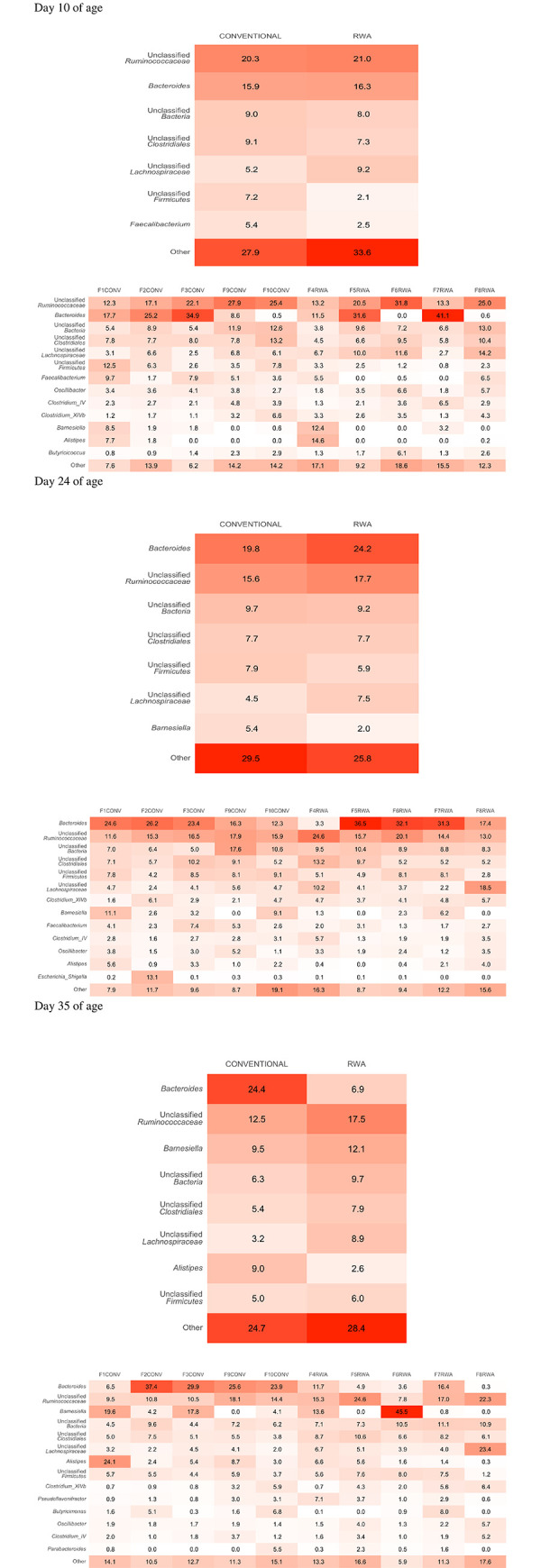
Heatmap comparing the relative abundance of difference in the genus level in the cecal microbiome of birds raised in the CONV and RWA groups during the growing phase: Starter (d 1 to 10 of age), grower (d 11 to 24 of age), and finisher (d 25 to 35 of age).

The heatmap of relative abundance of the microbial communities at the phylum level is shown in Fig 5 and the genus level is shown in Fig 6. On day 10 of age, the phylum Firmicutes was the most predominant phylum (67.1%) in both CONV and RWA chickens, followed by the phylum Bacteroidetes (21.9% in CONV and 23.50% RWA) (Fig 5). At the genus level, the genus unclassified *Ruminococcaceae* was the most predominant genus in both CONV (20.3%) and RWA chickens (21.0%), followed by the genus *Bacteroides* in CONV (15.9%) and RWA (16.3%) chickens (Fig 6).

On day 24, the phylum Firmicutes was the most abundant phylum in CONV (58.0%) and RWA (61.2%) chickens, followed by the phylum Bacteroidetes in CONV (29.1%) and RWA (28.3%) chickens (Fig 5). At the genus level, the relative abundance of the genus *Bacteroides* was lower in the CONV (19.8%) compared to RWA (24.2%) (Fig 6). However, the next most abundant genus was the genus unclassified *Ruminococcaceae*, which was lower in (CONV (15.6%) compared to RWA (17.7%) (Fig 6).

**Fig 7:**
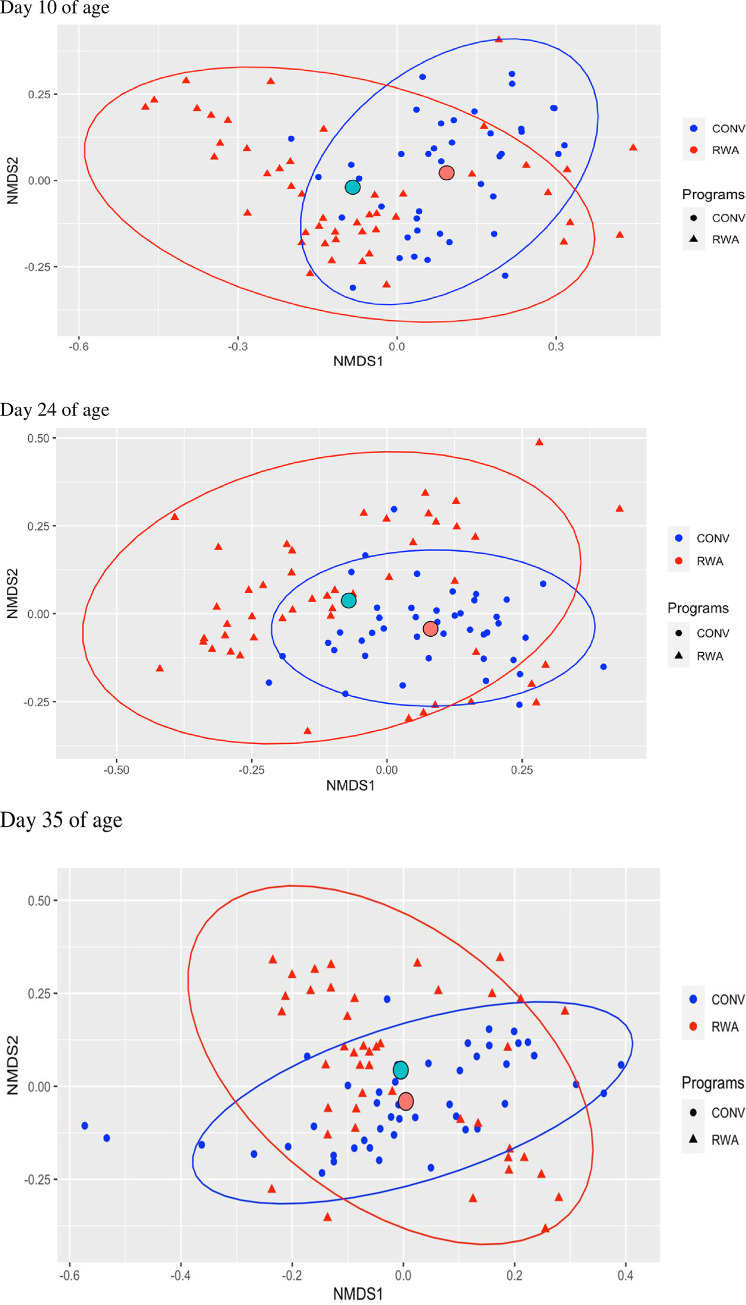
Compositional dissimilarity between CONV and RWA groups were calculated by the Bray-Curtis and used to make Non Metric Multidimensional Scaling (NMDS) plot illustrating the chicken cecal clustering by microbiota composition during the growing phase: Starter (d 1 to 10 of age), grower (d 11 to 24 of age), and finisher (d 25 to 35 of age).

**Fig 8:**
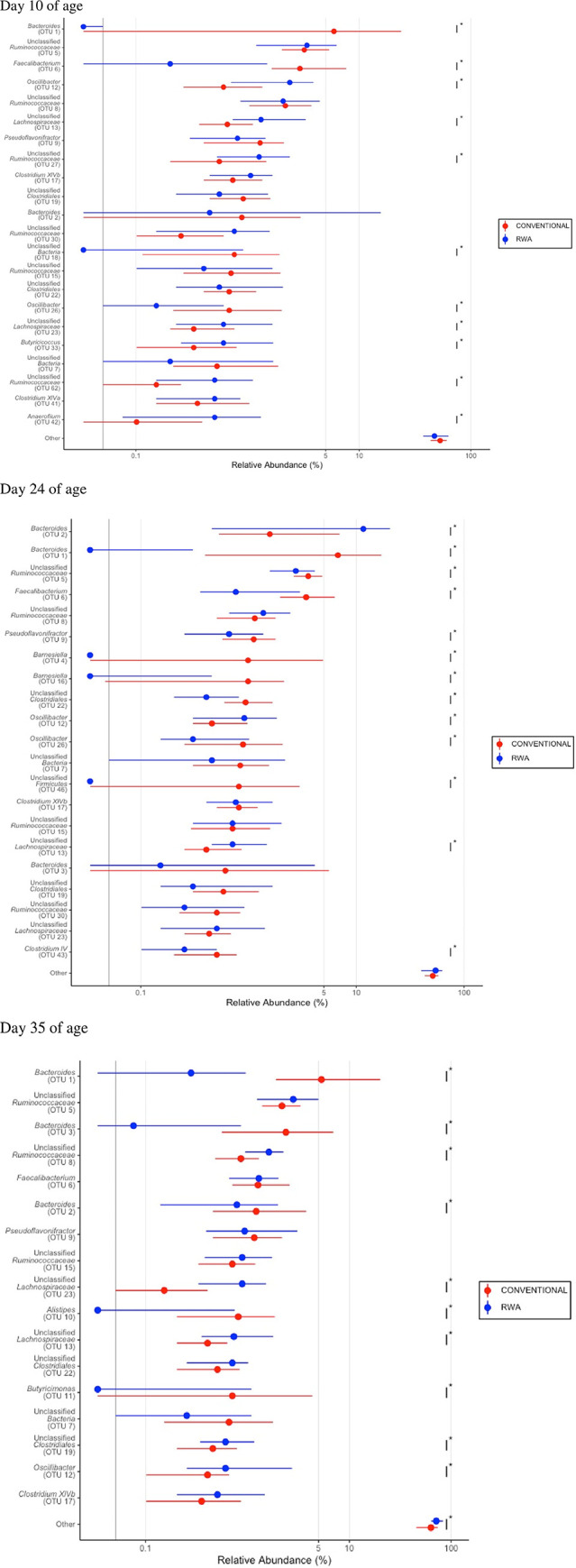
Differential enrichment of cecal microbiome analysis by DESeq2 on birds raised in the CONV and RWA groups on days 10, 24 and 35.

On day 35, the phylum Firmicutes was lower in CONV (42.3%) compared to RWA (63.7%), whereas phylum Bacteroidetes was more abundant in CONV (48.9%) than in RWA (25.1%) chickens (Fig 5). At the genus level, the genus *Bacteroides* was more abundant in CONV chickens (24.4%) compared to only 6.9% in RWA chickens, whereas the genus unclassified *Ruminococcaceae* was lower in CONV chickens (12.5%) compared to RWA chickens (17.5%) (Fig 6). Differences in cecal microbial composition between CONV and RWA chickens on days 10, 24, and 35 are depicted in Table 2. Beta diversity analysis using NMDS (Fig 7) based on PERMANOVA indicated a significant difference in the community structure of CONV and RWA farms on days 10 and 24 (Fig 8) (Table 2). Permutational analysis of multivariate dispersions (PERMDISP) tests with 9999 permutations results show that the dispersion of data points among CONV and RWA was significantly different on days 10 and 24 (Table 2).

**Table 2:** PERMANOVA and PERMDISP *p*-value of treatment groups on d 10, 24, and 35 (iterations = 1000).

Treatment	Adonis p-Value	PERMDISP p-Value
	Conventional	Conventional
Conventional-D10	-	-
Conventional-D24	-	-
Conventional-D35	-	-
RWA-D10	0.0009	0.013
RWA-D24	0.001	0.001
RWA-D35	0.147	0.142

### Differential enrichment of the cecal microbiome

Alpha diversity results indicated that birds in the CONV and RWA programs had significantly different microbiome structures. Next, we assessed the dissimilarity or similarity between microbial communities by identifying taxa exhibiting significantly different abundance between CONV and RWA groups.

DESeq2 analysis was used to compare differential enrichment of the cecal microbiome of CONV and RWA chickens on days 10, 24, and 35 of age (Fig 8). On day 10, the cecal microbiome of CONV chickens was enriched with genera *Bacteroides*, *Faecalibacterium*, and *Oscillibacter*, while the microbiome of RWA chickens was enriched with unclassified *Lachnospiraceae*, *Anaerofilum*, unclassified *Ruminococcaceae*, and *Butyricicoccus*.

**Fig 9:**
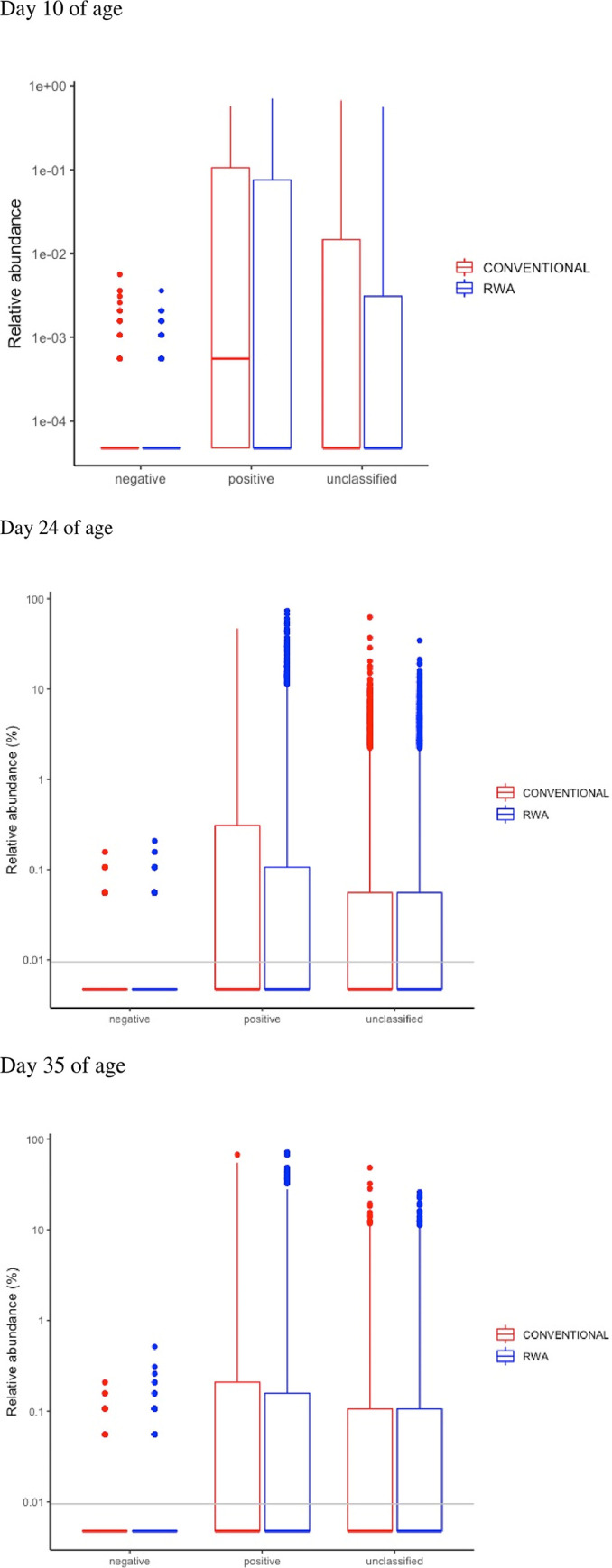
DESeq2 analysis illustrating dissimilarities in the cecal Gram-positive and negative microbiome of birds in the CONV and RWA groups during the growing phase: Starter (d 1 to 10 of age), grower (d 11 to 24 of age), and finisher (d 25 to 35 of age).

The cecal microbiome of CONV chickens had a significantly higher number of unclassified Gram-positive and -negative bacteria (Fig 9). On day 24, the cecal microbiome of CONV chickens was dominated with the genera *Bacteroides*, *Faecalibacterium*, *Pseudoflavonifractor*, unclassified *Clostridiales*, unclassified *Ruminococcaceae*, and *Clostridium_IV*, while the microbiome of RWA chickens was enriched with *Bacteroides*, *Oscillibacter* and family *Lachnospiraceae* (Fig 8). On day 35, the cecal microbiome of CONV chickens was significantly enriched with genera *Bacteroides*, *Alistipes* and *Butyricimonas*, while the microbiome of RWA chickens was dominated with genera unclassified *Ruminococcaceae*, unclassified *Lachnospiraceae*, unclassified *Clostridiale* and *Clostridium_XlVb* (Fig 8). There was no significant difference in the relative abundance of Gram-positive and -negative bacteria between the CONV and RWA chickens on days 24 and 35 of birds’ age (Fig 9).

## Discussion

A great deal of experimental evidence indicates that dietary antibiotics suppress the growth and proliferation of pathogenic bacteria, such as *C*. *perfringens*, either directly through destroying the bacterial cell wall and/or indirectly through modifying the gut microbiome composition. As the poultry industry is approaching a post-antibiotic era, there is an increasing emphasis on management strategies to maintain and promote gut health and the performance of broiler flocks. These strategies usually involve various feed and water supplements [reviewed by 19]. Indeed, the effects of different rearing management programs, including CONV and RWA, on growth performance and meat quality in broiler chickens have been investigated in both research and commercial settings [20]. Antibiotic effects on the gut microbiome composition and diversity have also been investigated in several research trials; however, whether the results of these studies reflect the changes in microbiome under field conditions remains to be investigated [20, 21].

Considering the important role of the intestinal microbiota in promoting host metabolism, maintaining intestinal immune homeostasis, and protecting against pathogen colonization, perturbation of the microbiome composition may lead to dysregulation of the metabolic process and the emergence of pathogenic microorganisms. As such, it is essential to understand how farming practices influence the composition and diversity of the chicken gut microbiome. In the current study, we aimed to investigate the diversity and differential enrichment of the cecal microbiome in chickens commercially reared under CONV and RWA programs.

In the current study, we aimed to investigate the diversity and differential enrichment of the cecal microbiome in chickens commercially reared under CONV and RWA programs. The alpha diversity analysis of the cecal microbiome indicated that birds reared under the CONV program had higher bacterial richness and diversity on days 10 and 24 than those raised under the RWA program. These results agree with [22], who observed that conventionally grown chickens had higher cecal richness, evenness, and diversity than those grown on the antibiotic-free farm. Similarly, a higher evenness and diversity were observed in the cecal microbiome of chickens treated with a cocktail of antibiotics compared to the control group that did not receive any antibiotics or feed additives [22]. Diverse factors, such as gut health management practices and chicken age and breed, can influence the microbiota diversity and population [20, 21, 23]. The notable increase in the cecal microbiome richness and diversity in the conventionally grown chickens on days 10 and 24 could be due to the elimination of the growth of microorganisms in a broad sense by antibiotics might have resulted in an increased abundance of nutrients and ecological niches for the growth of endogenous and exogenous microbes [24]. A surprising finding of this work was a shift in the microbiome diversity was observed on day 35 of age, with RWA chickens having a higher cecal diversity than the conventionally raised chickens, as previous studies has been shown that birds reared under the CONV program had higher bacterial richness and diversity [21].

The results of the present study indicated that CONV and RWA chickens have distinctly different cecal microbiomes. Consistent with the study of De Cesare [21] we observed a higher abundance of *Bacteroides* in conventionally raised chickens than in those raised without antibiotics. Similar observations have also been made by Chen [25], who reported a high relative abundance of genus *Bacteroides* in the cecal microbiome of chickens treated with virginiamycin. *Bacteroides* is a Gram-negative bacterium that plays a vital role in maintaining intestinal homeostasis and improving feed efficiency [26, 27] through metabolizing carbohydrates, including polysaccharides and oligosaccharides [28], and production of short-chain fatty acids, including acetate and propionate. We observed an increase in the relative abundance of *Butyricimonas*, which was found in a previous study to positively correlate with the growth performance of commercial broilers [29]. Taken together, the increased abundance of *Bacteroides* and *Butyricimonas* in conventionally grown chickens supports a role for antibiotic-associated changes in the cecal microbiome in improving growth performance traits, such as improving feed conversation ratio and increased body weight [30].

*Faecalibacterium* was among the most dominant genera in the microbiome of conventionally raised chickens. It is a Gram-positive butyrate-producing bacteria with documented probiotic properties and is regarded as a biomarker of a healthy gut [31]. A reduction in the abundance of *Faecalibacterium* was reported in chickens challenged with *C*. *perfringens* [32], suggesting the potential use of this bacterium as a feed additive to correct the *Clostridium*-induced dysbiosis and restore a healthy gut microbiome. In addition to their role in maintaining a healthy gut microbiome, *Faecalibacterium* possesses other benefits to the host, including regulation of the lipid metabolism and glucose homeostasis in broilers [26] and increasing broiler production efficiency through improving feed conversation ratio and reducing feed intake [32].

The genus *Oscillibacter* was among the taxa exhibiting significant differential abundance in the cecal microbiome of the CONV chickens. It has been previously shown that *Oscillibacter* bacteria contribute to butyrate production by the gut microbiome by expressing acetyl-CoA acetyltransferase, a key enzyme in the acetyl-coenzyme a (CoA) pathway, which is one of the two pathways involved in butyrate synthesis by butyrate-producing bacteria [33]. Furthermore, a reduction in the relative abundance of genus *Oscillibacter* was reported in *Eimeria*-challenged broiler chickens, which reveals the indirect role of antibiotics in enhancing host resistance to not only bacterial but also parasitic pathogens through modulating the gut microbiome [34].

Consistent with the previous observation made by Robinson [35] that members of the family Clostridiaceae, including *Clostridium* IV and XIVa, were preferentially enriched in the cecal microbiota of the AGPs-supplemented chickens, we also demonstrated a significant increase in the abundance of genera *Clostridium* IV and XlVb in the cecal microbiome of CONV chickens. *Clostridium* IV is a butyrate-producing bacterium, while *Clostridium*_XlVb plays an essential role in breaking down various indigestible polysaccharides, such as cellulose and hemicelluloses [36]. Another observation was that the microbiome of the CONV chickens was enriched with the genus *Alistipes* (a member of phylum Bacteroidete), which is also essential for the breakdown of indigestible polysaccharides in the intestine [37].

Supplementing chickens with butyrate was found to prevent intestinal inflammation by inducing the production of immunoregulatory cytokines such as interleukin (IL)-10. A previous study in mice demonstrated that increasing the abundance of cluster *Clostridium* IV bacteria in the mouse intestine can mitigate ulcerative colitis by inducing the production of transforming growth factor (TGF)-β, which, in turn, enhances the expansion and differentiation of T regulatory (Treg) cells [38]. It is, therefore, conceivable that supplementation of these specific members of bacteria to chickens raised without antibiotics may help regulate the intestinal immune system, thereby preventing intestinal inflammation caused by a proliferation of pathogenic bacteria, such as *C*. *perfringens* and limiting the resources such as food, space, or nutrients for the overgrowth of pathogenic bacteria.

A higher relative abundance of genus *Pseudoflavonifractor* was observed in CONV chickens compared to RWA chickens. These results are consistent with Yitbarek [22] who also observed that chickens fed with a cocktail of antibiotics had a high relative abundance of *Pseudoflavonifractor* in their cecal microbiome. The authors also found a correlation between the abundance of *Pseudoflavonifractor* and the expression of interleukin 22 in the cecal tonsils, a cytokine that possesses pro- and anti-inflammatory properties and plays an important role in inducing cellular proliferation and tissue protection and regeneration [39]. These findings highlight the beneficial role of dietary antibiotics in modifying the gut microbiota and increasing the production of microbiota-derived metabolites, which contribute to improved nutrient utilization and regulation of the intestinal mucosal immune function.

Compared to the conventionally grown chickens, a different composition of the cecal microbiome was observed in the chickens reared on RWA programs. The cecal microbiome of RWA chickens was enriched with the family *Lachnospiraceae*, which contributes to the fermentation of complex polysaccharides and the production of short-chain fatty acids, including acetate, butyrate, and propionate, which serve as a source of energy for the intestinal epithelial cells [40], in addition to their health-promoting and anti-inflammatory effects. *Lachnospiraceae* and *Ruminococcaceae* belong to the order *Clostridiales*, which comprise a significant proportion of taxonomic groups in the healthy chicken and mammalian hindgut [40, 41]. Previous studies reported a depletion of *Lachnospiraceae* in the chickens supplemented with dietary fishmeal as a predisposing factor for necrotic enteritis [42], indicating an association between the abundance of *Lachnospiraceae* and the occurrence of necrotic enteritis. Robinson [35] have also found that birds supplemented with antibiotics and ionophores had a lower abundance of *Lachnospiraceae* species, which is consistent with our findings.

Furthermore, the family *Ruminococcaceae*, a major butyrate producer known for its role in maintaining gut health, was the most dominant family in the chickens read on the RWA program. A reduction in the abundance of *Ruminococcaceae* strains was observed in the birds with necrotic enteritis [41], highlighting the importance of increasing the abundance of this family to prevent enteric infection with opportunistic pathogens, such as *C*. *perfringens*.

In this study, *Butyricicoccus*, which belongs to the family *Ruminococcaceae*, was more abundant in the chicken cecal microbiome in the RWA chickens. *Butyricicoccus* is a butyrate-producing bacteria and was also found to play a vital role in nutrient digestion and absorption [43]. A previous study in humans reported that patients suffering from inflammatory bowel disease (IBD) had a significantly lower relative abundance of the genus *Butyricicoccus* compared to healthy people [44]. Similarly, the relative abundance of genus *Anaerofilum* which produces short-chain fatty acids was significantly higher in the cecal microbiome of RWA chickens than in CONV chickens [45]. These results align with the observations from our earlier study and those of others that feeding anticoccidial and antibiotics to chickens was associated with a significant decrease in the relative abundance of *Anaerofilum* in their cecal microbiota [13, 46].

Altogether, manipulation of the gut microbiome via feed additives is warranted to increase the microbial diversity in the RWA and, more specifically, to increase the abundance of beneficial gut bacteria, including members of Phyla Firmicutes and Bacteroidetes.

## Conclusions

In the present study, we sought to profile and compare the cecal microbiome of commercial broilers reared under different management practices. The cecal microbiome of CONV chickens tended to have greater microbiome diversity and an increased enrichment with beneficial microbial communities with high functional redundancy compared to RWA chickens. These findings provide a foundation for further research on tailoring feed formulation or developing a microbial consortium to modify the composition of the gut microbiome of RWA chickens.
