# Correction: Intestinal microbiome profiles in broiler chickens raised without antibiotics exhibit altered microbiome dynamics relative to conventionally raised chickens

**DOI:** 10.1371/journal.pone.0304225

**Published:** 2024-05-17

**Authors:** Seyed Hossein Karimi, Khaled Abdelaziz, Heidi Spahany, Jake Astill, David Trott, Blake Wang, Alice Wang, John Parkinson, Shayan Sharif

The first author’s name is spelled incorrectly. The correct name is: Seyed Hossein Karimi. The correct citation is: Karimi SH, Abdelaziz K, Spahany H, Astill J, Trott D, Wang B, et al. (2024) Intestinal microbiome profiles in broiler chickens raised without antibiotics exhibit altered microbiome dynamics relative to conventionally raised chickens. PLoS ONE 19(4): e0301110. https://doi.org/10.1371/journal.pone.0301110.

There are errors in the Author Contributions. The correct contributions are:

**Conceptualization:** Seyed Hossein Karimi, Shayan Sharif.

**Data curation:** Seyed Hossein Karimi.

**Formal analysis:** Seyed Hossein Karimi.

**Investigation:** Seyed Hossien Kairmi, Khaled Abdelaziz, Heidi Spahany, Jake Astill, David Trott, Blake Wang, Alice Wang, John Parkinson, Shayan Sharif.

**Methodology:** Seyed Hossein Karimi, Shayan Sharif.

**Project administration:** Seyed Hossein Karimi, Shayan Sharif.

**Resources:** Shayan Sharif. **Supervision:** Shayan Sharif

**Validation:** Seyed Hossein Karimi, Shayan Sharif.

**Visualization:** Seyed Hossein Karimi.

**Writing–original draft:** Seyed Hossein Karimi.

**Writing–review & editing:** Seyed Hossein Karimi, Shayan Sharif.
